# Dynamic changes of blood glucose, serum biochemical parameters and gene expression in response to exogenous insulin in Arbor Acres broilers and Silky fowls

**DOI:** 10.1038/s41598-020-63549-9

**Published:** 2020-04-21

**Authors:** Jiefei Ji, Yafei Tao, Xiangli Zhang, Jiajia Pan, Xinghao Zhu, Huanjie Wang, Pengfei Du, Yao Zhu, YanQun Huang, Wen Chen

**Affiliations:** College of Animal Science, Henan Agricultural University, Zhengzhou, Henan P.R. China

**Keywords:** Biochemistry, Genetics

## Abstract

Silky chicken is a breed of chickens with black skin and slow growth rate used in Chinese traditional medicine, whereas Arbor Acres broiler is a well-known commercial breed in the poultry industry, it is featured by a large size, rapid-growth rate, high feed-conversion rate and strong adaptability. The difference in their rate of growth may be primarily related to different mechanism for glucose metabolism. Here we compared the insulin sensitivity of the two breeds; we investigated the temporal changes (at 0 min, 120 min and 240 min) of serum insulin and other biochemical parameters and determined the spatio-temporal changes of gene mRNA abundance in response to exogenous insulin (80 μg/kg body weight). The results indicated that: (1) Silky chickens showed stronger blood glucose recovery than broilers in the insulin resistance test. (2) The serum urea level in Silky chickens was twice of broilers; exogenous insulin significantly up-regulated serum uric acid level in Silky fowls in a time-dependent manner and increased serum cholesterol content at 120 min. (3) Two breeds showed distinctly different temporal changed in serum insulin in response to exogenous insulin stimulation. The fasting serum insulin concentration of broilers was three-fold of Silky chickens at the basal state; it decreased significantly after insulin injection and the levels at 120 min and 240 min of broilers were only 23% (*P* < 0.01) and 14% (*P* < 0.01) of the basal state, respectively. Whereas the serum insulin content in Silky chickens showed stronger recovery, and the 240 min level was close to the 0 min level. (4) GLUT2, GLUT12, neuropeptide Y and insulin receptor (IR) were predominantly expressed in the liver, pectoralis major, olfactory bulb and pancreas, respectively, where these genes presented stronger insulin sensitivity. In addition, the IR mRNA level was strongly positively with the GLUT12 level. In conclusion, our findings suggested that Silky chickens have a stronger ability to regulate glucose homeostasis than broilers, owing to their higher IR levels in the basal state, stronger serum insulin homeostasis and candidate genes functioning primarily in their predominantly expressed tissue in response to exogenous insulin.

## Introduction

Insulin is secreted by islet beta cells^1^, and is the only protein hormone in the body that can lower blood glucose levels^2^. The action of insulin are a critical part of normal development, food intake, and energy balance^3^. Chickens are insulin-resistant and significantly resistant to high concentrations of insulin; they have higher blood glucose concentration than mammals, even in a fasted state^4^, but do not develop diabetes. The uptake of glucose in the bloodstream is the rate-limiting step in systemic glucose utilization, and this process is regulated by the membrane protein family of glucose transporters (GLUTs)^5^. The expression and protein activity of GLUTs are important for maintaining glucose homeostasis and providing nutrient substrates^6^. GLUT4 is present almost exclusively in insulin-sensitive tissues such as muscle and adipose tissue, and it is an insulin sensitive glucose transporter responsible for rapid glucose transport following insulin stimulation in mammals^7,8^. However, chickens were thought to lack GLUT4^9,10^, and the chicken insulin signaling cascade appears to be different from that in mammals^11,12^. Despite this, insulin-dependent glucose transport may still be functional in birds^13^. It has been found that other GLUT proteins may respond to insulin and promote glucose uptake when tissues are stimulated by insulin in birds. GLUT2 has a low affinity and high capacity for glucose transport, plays an important role in the body’s glucose metabolism and regulates glucose homeostasis in mammals^14^. GLUT2 is abundantly expressed in chicken liver, an important tissue that responds to insulin^14^. The relatively high GLUT2 expression in liver might be one of the clues for understanding chicken glucose homeostasis that sustains hyperglycemia. In addition, GLUT12 may be a novel insulin-sensitive GLUT which has been described to act as an insulin-sensitive GLUT in mammals and function similar to GLUT4^11^.

Zhang *et al*. have studied the effects of insulin injection (at 60 min) on the mRNA levels of glucose transporter and appetite-associated factors in various tissues of chickens selected for low or high body weight^14^. Franssens *et al*. have reported the effects of insulin on plasma glucose concentrations and the expression of hepatic glucose transporters and key gluconeogenic enzymes during the perinatal period in broiler chickens^12^. Coudert *et al*. have determined the expression of GLUT1, GLUT8 and GLUT12 in different chicken muscles during ontogenesis, and found that the expression of GLUT12 differs considerably among muscles but is not necessarily associated with the muscle contractile or metabolic type^15^. Insulin immuno-neutralization decreases food intake in chickens without altering the expression of hypothalamic transcripts involved in food intake and metabolism^16^.

The olfactory bulb (OB), located in the forebrain of vertebrates, receives neural input regarding odors detected by cells in the nasal cavity, which contains the highest levels of insulin and insulin receptors (IRs) in the brain^17^. Insulin has also been reported to be involved in the modulation of olfactory function in rats. These findings suggest that the OB should have important functions in regulating energy homeostasis. The response of the OB to exogenous insulin was previously unknown in chickens.

In addition to stimulating postprandial glucose uptake, an another major mechanism of insulin action is in stimulating FFA (Free Fat Acid) esterification^18^. Insulin-resistant states, such as obesity and type 2 diabetes, are frequently exhibit altered lipoprotein levels and composition^19^, one important complication is an atherogenic dyslipidemia profile characterized by hypertriglyceridemia, low plasma high-density lipoproteins (HDL) cholesterol and a small, dense low-density lipoprotein (LDL) particle profile^20,21^. In other words, insulin resistance is also closely related to lipid metabolism.

Moreover, the insulin-dependent effects on gene expression in chickens are unclear. There have been no reports on the temporal changes in circulating metabolites and glucose-metabolism related genes in chickens under insulin stimulation. Silky chickens are used in Chinese traditional medicine and have black skin and low growth rates, whereas Arbor Acres (AA) broilers have rapid early growth rates. Here, we focused on revealing the dynamic responses to exogenous insulin in the breeds by comparing the insulin sensitivity through insulin resistance tests, investigating the changes in circulating insulin and metabolite levels, and determining the mRNA levels of GLUT2, GLUT12, IR and neuropeptide Y (NPY) in insulin-sensitive tissues including the liver, pectoralis muscle (PM), pancreas and OB. These results provide new insights to improve understanding the fine regulation of glucose utilization in chicken tissues. In addition, broilers may be a good model of human obesity, and research using this model would provide a reference for understanding human health.

## Results

### Insulin resistance of AA and Silky

**Figure 1:**
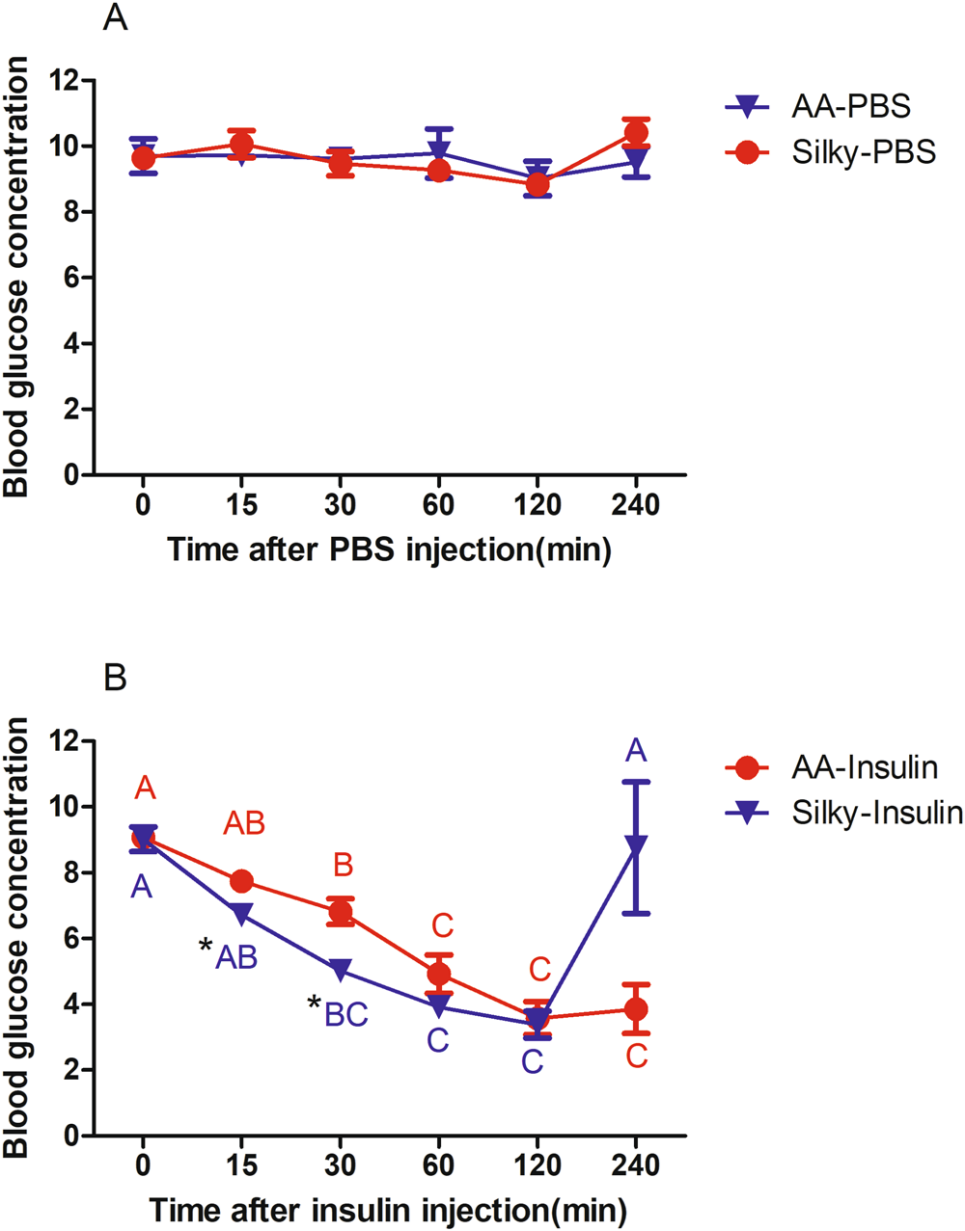
Insulin tolerance test of AA broilers and Silky fowls. (**A**) Control groups (n = 6/breed) were injected with an equal volume of PBS after 12 h fasting. (**B**) Experimental groups (n = 6/breed) received an injection of insulin (80 μg/kg BW). In the same breed, different letters indicate *P* < 0.05, and the same letter indicates *P* > 0.05. In the same time point, * means *P* < 0.05 between two breeds, no * means *P* > 0.05.

The blood glucose concentration was stable at approximately 10 mmol/L within 240 min after PBS injection in both broilers and Silky fowls (Fig. 1A). After insulin injection (Fig. 1B), the blood glucose concentration decreased almost linearly in the first 120 min, and the blood glucose concentration in Silky fowls decreased more severely. Among them, the blood glucose of Silky fowls was significantly lower than that of broilers at 15 min (*P* < 0.05) and 30 min (*P* < 0.05) after insulin injection. At 120 min after insulin injection, the blood glucose of broilers and Silky fowls decreased to a similar level (approximately 4 mmol/L). Immediately after measuring the blood glucose of 120 min post insulin injection, restore feeding to chickens for 2 h and measured the blood glucose at last. The blood glucose concentration of Silky fowls increased rapidly, and the 240 min level was close to 0 min level (*P* > 0.05), whereas the blood glucose of broilers increased less intensely after 2 h refeeding, and the 240 min level remained close to the 120 min level (*P* > 0.05). At 240 min, Silky fowls had higher blood glucose than AA broilers (*P* = 0.058, Fig. 1B).

### Effects of exogenous insulin on serum insulin concentration

**Figure 2:**
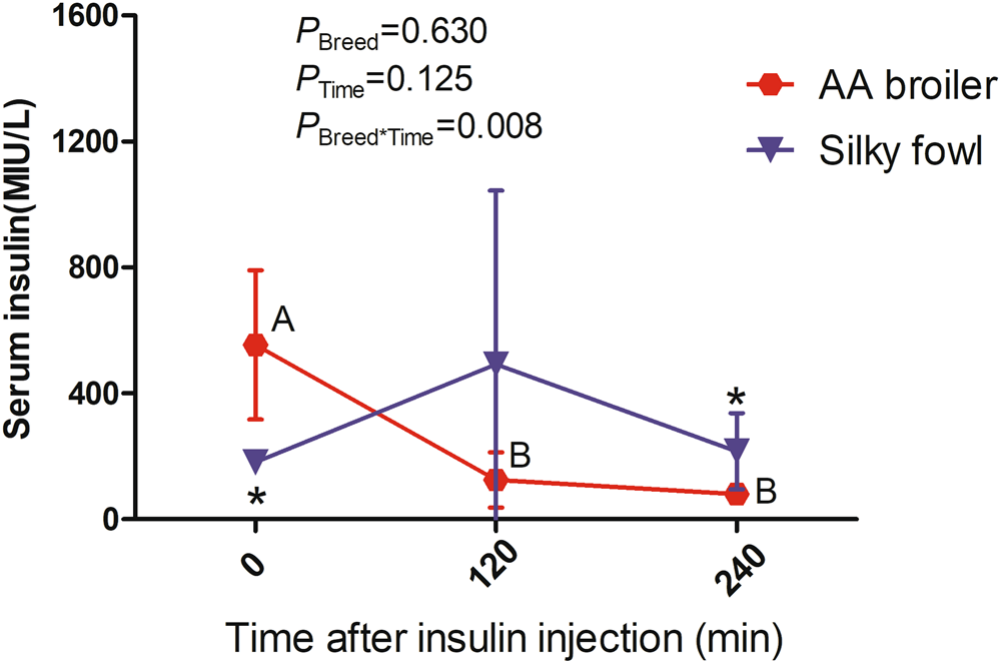
The effect of exogenous insulin on serum insulin level. Birds were received 80 μg insulin/kg BW after 12 h fasting. Different capital letters across time indicates *P* < 0.05 in the same breed. * across breed indicates *P* < 0.05. Absence of letter/* or same letter indicates *P* > 0.05. The effect values for two-way ANOVA were presented in Figure.

Based on the data of the effect of different insulin treatment time (0 min, 120 min and 240 min, denoted the time effect) on broilers and Silky fowls, two-way ANOVA was first performed to analyze the effect of breed and time effect on the serum insulin levels. The effect of exogenous insulin on the serum insulin concentration showed a clear interaction of time and breed (*P* = 0.008). The dynamic changes of serum insulin showed a significant difference between Silky fowls and AA broilers in response to exogenous insulin. Therefore, we analyzed the effect of breed and time effect on the serum insulin levels separately by one-way ANOVA. The serum insulin levels of AA broilers were approximately three-fold higher than those of Silky fowls at the basal state (12 h fasting, Fig. 2, *P* < 0.05). After insulin injection, the serum insulin concentration of broilers markedly decreased, the insulin level at 120 min and 240 min were only 23% (*P* < 0.05) and 14% (*P* < 0.05) of that at the basal state, respectively. In contrast, Silky fowls showed stronger recovery of serum insulin levels after exogenous insulin stimulation; the serum insulin levels fluctuated and increased somewhat at 120 min (*P* > 0.05), but returned to the 0 min level at 240 min (Fig. 2). Besides, at 120 min and 240 min after insulin stimulation, the serum insulin concentration of broilers were lower than that in Silky chickens, and the difference reached a significant level at 240 min (*P* < 0.05).

### Effects of exogenous insulin on serum biochemical indexes

**Figure 3:**
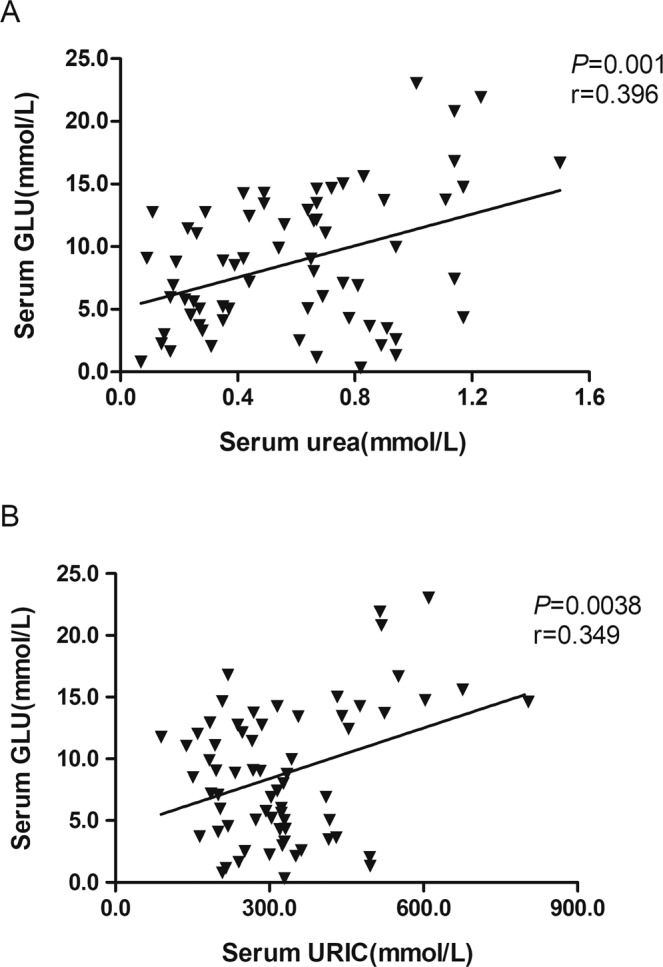
The correlation analysis for serum glucose with other biochemical indexes. **(A**) Correlation of serum GLU with urea (n = 67). (**B**) Correlation of serum GLU with URIC (n = 67).

We evaluated the effects of insulin treatment time (0 min, 120 min and 240 min) and breed on serum biochemical indexes through one-way ANOVA and two-way ANOVA (Table 2). The serum urea content was significantly (*P* < 0.05) higher in Silky fowls than AA broiler chickens, whereas exogenous insulin had no significant effect on serum urea level. The interaction of breed and time effect was significant for serum URIC level (*P* < 0.05), exogenous insulin did not significantly affect serum URIC level in broilers, whereas it significantly up-regulated the URIC level in Silky fowls in a time dependent manner. The 240 min URIC level of Silky fowls was significantly higher than the 0 min level (before insulin injection) of Silky fowls and the 240 min level of broilers (*P* < 0.05). In addition, there was a significant interaction of breed and time effect on serum CHOL (*P* < 0.05). Exogenous insulin increased the serum CHOL at 120 min of Silky chickens, which was significantly higher than that of Silky fowls at 0 min and 240 min and broilers at 120 min post insulin injection. No significant effects were observed on other serum lipid indexes, including TG, HDLD and LDLD (Table 2). Furthermore, the correlation analysis between serum glucose and other serum biochemical indexes showed that serum glucose was significantly correlated with urea (Fig. 3A, r = 0.396, *P* = 0.001) and URIC content (Fig. 3B, r = 0.349, *P* = 0.0038).

**Table 1:** Primers used for real-time PCR.

Gene	Acession No.	Sequence(forward/reverse, 5′-3′)
GLUT2	NM_207178.1	AGTACATCGCGGATCTGTGC/TGACTTCCCCTTCGTTTCGG
GLUT12	XM_419733.4	TGGGGTCTCACACAGAGAGT/GGACGAGCCAAGACATTGGT
NPY	M87294.1	GATCCCGGTTTGAAGACCCT/CTGCATGCACTGGGAATGAC
IR	XM_001233398.4	CAGTGATGTGTACGTTCCCGA/CCAGCTCTCCCTTCACGATG

IR = insulin receptor; NPY= neuropeptide Y.

**Table 2:** Effect of insulin treatment on serum biochemical indicators.

	Items Time point^a^	Breed		P Value		
		AA broiler	Silky fowl	Breed	Time	Breed×Time
UREA mmol/l	0 min	0.41 ± 0.08	0.75 ± 0.07	#	NS	NS
	120 min	0.24 ± 0.04*	0.85 ± 0.08			
	240 min	0.31 ± 0.04*	0.82 ± 0.13			
URIC mmol/l	0 min	238.00 ± 61.05	230.67 ± 16.74 A	#	NS	#
	120 min	334.25 ± 58.04	359.50 ± 56.57			
	240 min	197.60 ± 17.83*	482.33 ± 74.33B			
CHOL mmol/l	0 min	3.10 ± 0.21	3.52 ± 0.20 A	#	#	#
	120 min	3.22 ± 0.37*	4.78 ± 0.50B			
	240 min	3.27 ± 0.10	3.02 ± 0.24 A			
TG mmol/l	0 min	0.26 ± 0.03	0.41 ± 0.04	NS	NS	NS
	120 min	0.38 ± 0.04	0.61 ± 0.14			
	240 min	0.43 ± 0.10	0.47 ± 0.06			
HDLD mmol/l	0 min	2.33 ± 0.13	2.45 ± 0.15	NS	NS	NS
	120 min	2.20 ± 0.28	2.45 ± 0.32			
	240 min	2.12 ± 0.21	2.13 ± 0.16			
LDLD mmol/l	0 min	0.63 ± 0.06	0.83 ± 0.19	NS	NS	NS
	120 min	0.75 ± 0.17	1.89 ± 0.79			
	240 min	0.75 ± 0.09	0.70 ± 0.07			

^a^Means at 0 min, 120 min and 240 min after insulin injection (denoted the time effect). Data are expressed as mean ± standard error. Different capital letters across time indicates *P* < 0.05, * across breed indicates *P* < 0.05, absence of letter or * indicates that the statistical analysis was non-significant. NS = not significant, ^#^ means *P* < 0.05.

### The mRNA abundance of candidate genes in two breeds

**Figure 4:**
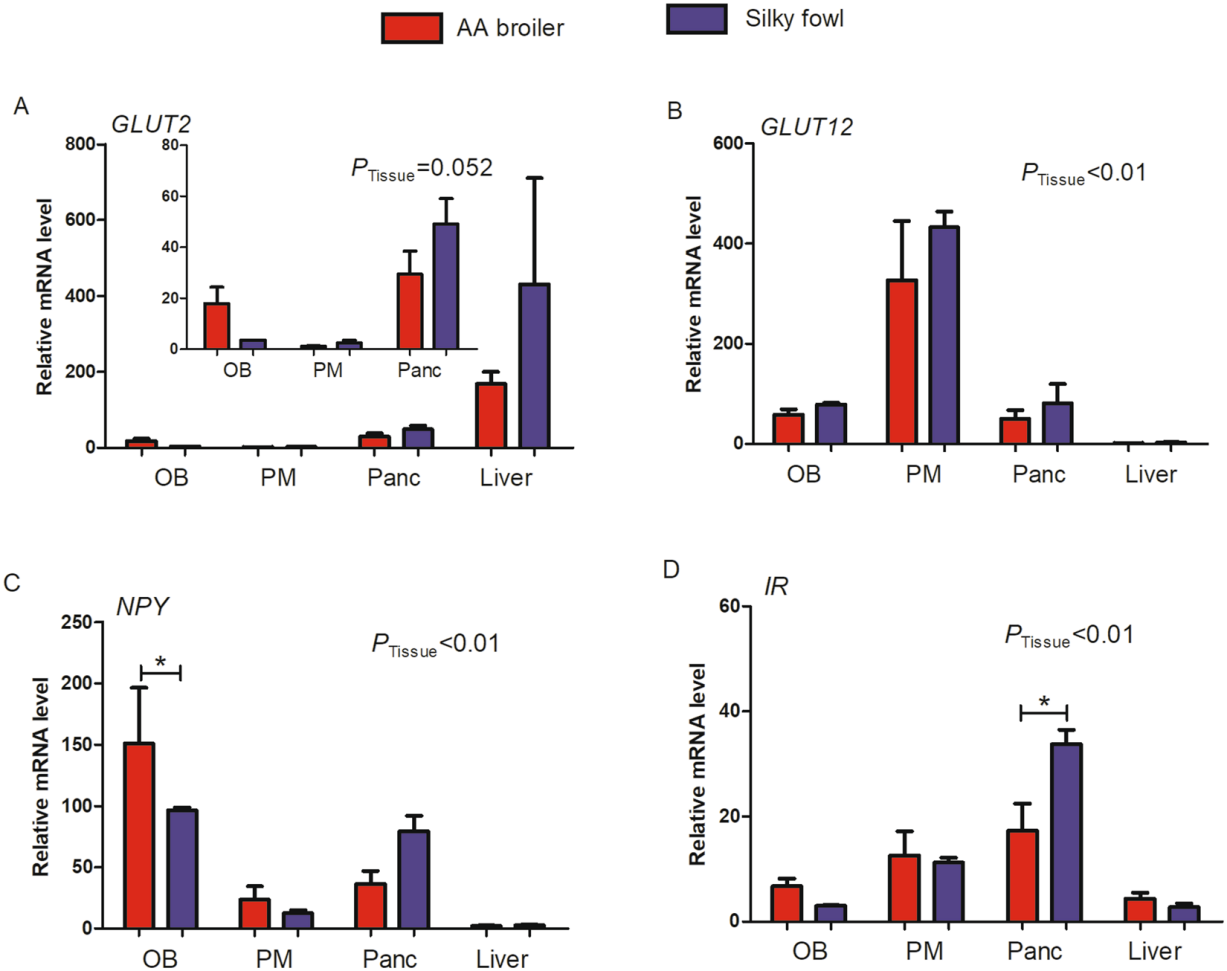
The mRNA abundance of candidate genes among different tissues in the basal state. Chickens were slaughtered after 12 h fasting (n = 6/breed). (**A**) GLUT2; (**B**) GLUT12; (**C**) NPY; (**D**) IR. OB = olfactory bulb, PM = pectoralis major, Panc = pancreas. *above bars indicates significant difference at *P* < 0.05.

Real-time PCR was used to quantitatively analyze the abundance of GLUT2, GLUT12, NPY and IR mRNA in four different tissues including the OB, PM, pancreas and liver of 47 d female AA broilers and Silky fowls. Based on the data from four tissues of broilers and Silky fowls, one-way ANOVA was used to analyze the effect of tissue. At the basal state of 47 d, the mRNA level of GLUT2, GLUT12, NPY and IR varied significantly among tissues, and each gene was clearly predominantly expressed in a different tissue (Fig. 4). The mRNA abundance of GLUT2 was the greatest in the liver (*P* = 0.052, Fig. 4A); GLUT12 was predominantly expressed in the PM (*P* < 0.01, Fig. 4B); NPY mRNA level was the highest in the OB (*P* < 0.01, Fig. 4C) and the highest IR mRNA level was found in the pancreas (*P* < 0.01, Fig. 4D) among the four tissues. Moreover, we analyzed the effect of breed on gene mRNA abundance in a certain tissue by one-way ANOVA, the effect of breed on mRNA levels was significant only for NPY in the OB (AA broilers > Silky fowls, *P* < 0.05, Fig. 4C) and IR in the pancreas (AA broilers < Silky fowls, *P* < 0.05, Fig. 4D).

### Effects of exogenous insulin on mRNA levels of candidate genes

Real-time PCR was used to quantitatively analyze the variation of GLUT2, GLUT12, NPY and IR mRNA abundance in four different tissues of 47 d female AA broilers and Silky fowls after insulin injection. Based on the data from four tissues (OB, PM, pancreas and liver) from broilers and Silky fowls of different insulin treatment time (0, 120, 240 min), two-way ANOVA was performed to analyze the effect of breed and time on these four gene mRNA abundance. This analysis (Table 3) showed that each gene was insulin sensitive in its predominantly expressed tissue under exogenous insulin stimulation, and a significant main effect of time (after insulin injection) was observed at the mRNA level for four candidate genes: GLUT2 (*P* = 0.029), GLUT12 (*P* = 0.050), NPY (*P* = 0.010) and IR (*P* = 0.013). In addition, the mRNA level of GLUT2, NPY and IR showed a significant interaction of breed and time effect in the OB response to exogenous insulin, whereas the interaction did not significantly affect the expression of these four genes in the liver, PM and pancreas (Table 3).

**Table 3:** Effect of insulin treatment on genes of broilers and Silky fowls.

	P value	Olfactory bulb	Pectoralis major	Pancreas	Liver
GLUT2	Breed	<0.01	0.266	0.845	0.147
	Time^a^	0.001	0.330	0.014	0.029
	Breed*Time	<0.01	0.909	0.112	0.235
GLUT12	Breed	0.34	0.506	0.050	0.762
	Time	0.984	0.050	0.409	0.152
	Breed*Time	0.161	0.742	0.890	0.117
NPY	Breed	0.499	0.546	0.331	0.122
	Time	0.010	0.027	0.582	0.504
	Breed*Time	0.006	0.747	0.266	0.509
IR	Breed	0.697	0.077	0.002	0.073
	Time	0.649	0.754	0.013	0.058
	Breed*Time	0.035	0.232	0.068	0.322

^a^Time effect means the different time point post insulin treatment.

**Figure 5:**
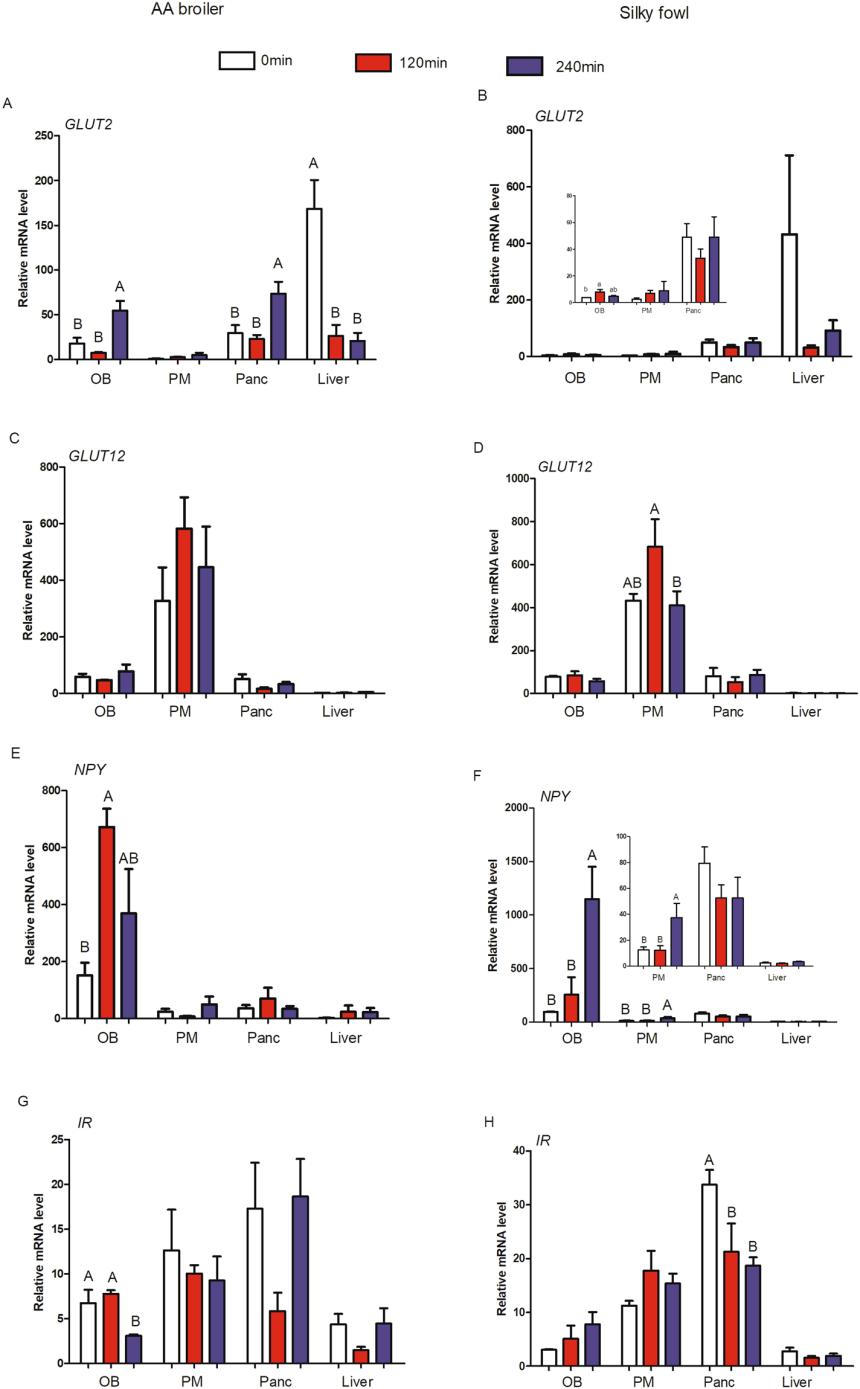
The effects of exogenous insulin on mRNA abundance of candidate genes among different tissues. Fowls were injected with 80 μg insulin/kg BW via hypodermic (n = 6 for each time-point/breed). Left side is for AA broilers and right side is for Silky fowls. (**A**,**B**) GLUT2; (**C**,**D**) GLUT12; (**E**,**F**) NPY; (**G,H**) IR. OB = olfactory bulb; PM = pectoralis major; Panc = pancreas. Different capital letter across samples in one tissue indicates *P* < 0.05, same letter indicates *P* > 0.05.

In addition, we analyzed the effect of insulin treatment time on gene mRNA abundance of AA broilers or Silky fowls separately by one-way ANOVA (Fig. 5). After insulin injection, the abundance of GLUT2 mRNA decreased in the liver of broilers (Fig. 5A) and Silky chickens (Fig. 5B), and the difference reached a statistical level in broilers (*P* < 0.05). In addition, at 240 min post insulin injection, GLUT2 mRNA level was significantly higher than the 0 min and 120 min in the OB and pancreas of broilers (Fig. 5A), whereas GLUT2 mRNA level was not significantly different among time points after insulin injection in these four tissues of Silky chickens (Fig. 5B). These results indicated that GLUT2 is more insulin sensitive in broilers than in Silky chickens (Fig. 5A,B). GLUT12 was predominantly expressed in the PM, where it was up-regulated (*P* = 0.05) at 120 min, and returned to the basic level at 240 min after exogenous insulin stimulation both in AA broilers and Slky fowls (Fig. 5C,D). NPY was predominantly expressed in the OB, where its dynamic changes showed clear breed-specific differences under insulin stimulation. In AA broilers, the mRNA abundance of NPY in the OB increased approximately four-fold at 120 min (*P* < 0.05) and then slightly decreased at 240 min in response to exogenous insulin (Fig. 5E). In contrast, in Silky fowls, NPY mRNA showed a slight increase at 120 min and then a drastic up-regulation (approximately five-fold) within 120–240 min (*P* < 0.05) after insulin stimulation (Fig. 5F). In addition, the NPY mRNA levels in Silky chickens were also significantly up-regulated in PM tissues, in a pattern similar to that in OB tissues (*P* < 0.05, Fig. 5F). In response to exogenous insulin, IR mRNA expression also showed different change patterns (*P*_Breed*Time_ = 0.068, Table 3) in the pancreas and OB tissues (*P*_Breed*Time_ = 0.035, Table 3) between broilers and Silky chickens. After insulin injection, there were no significant change occurred at 120 min of the IR mRNA level but it were significantly down-regulated at 240 min in the OB (*P* < 0.05) of broilers and dropped to approximately half of the previous levels (Fig. 5G); while in the OB of Silky fowls, the IR mRNA abundance showed a rising trend in response to exogenous insulin but not reach the statistical levels (*P* > 0.05). The IR mRNA level was significantly down-regulated (*P* < 0.05) at 120 min and 240 min in the pancreas of Silky chickens and there were no significant difference between 120 min and 240 min (Fig. 5H), but there were no significant difference among three time points in the pancreas of Broilers (Fig. 5G).

**Figure 6:**
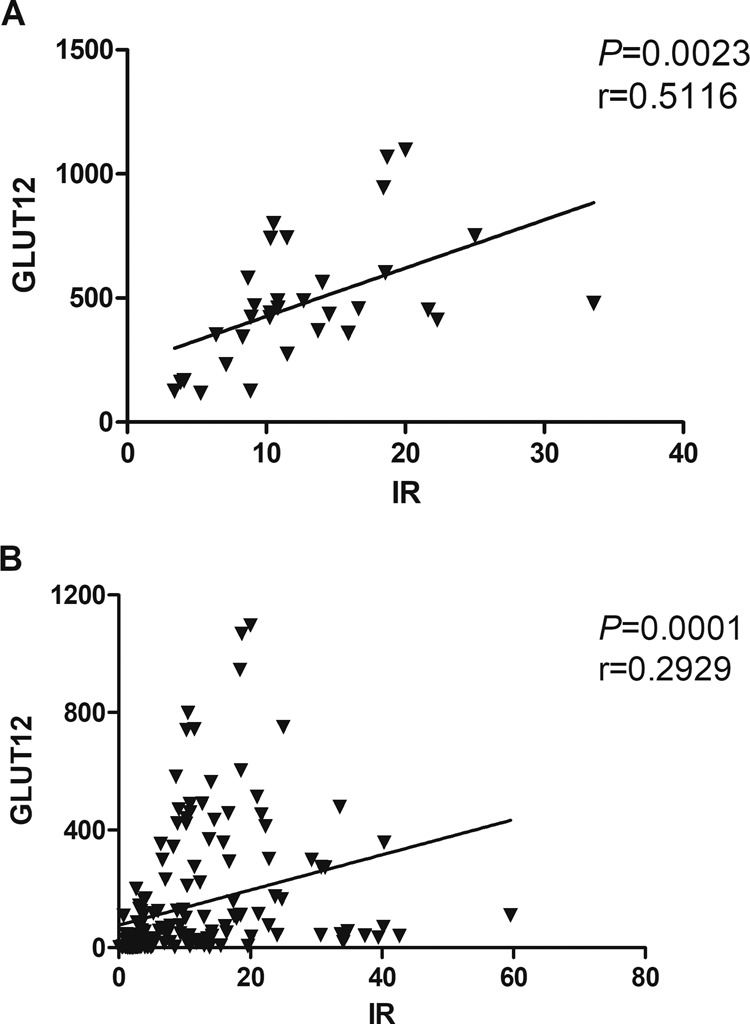
The correlation of GLUT12 and IR mRNA abundance. In pectoralis muscle (n = 36); (**B**) In all detected samples (n = 144).

Based on the data of four genes mRNA abundance in four tissues (OB, PM, pancreas and liver) of 47 d broilers and Silky fowls, pearson correlation between the mRNA levels of genes was analyzed. The results revealed that there was a strong positive correlation between the mRNA levels of IR and GLUT12 in PM tissue (Fig. 6A, r = 0.5116, *P* = 0.0023) and all detected samples (Fig. 6B, r = 0.2929, *P* = 0.0001).

## Discussion

Many homeostatic events are maintained in animal bodies, for example, the blood glucose level is under persistent tight regulation because of its crucial physiologic aspects, given that too much or too little glucose can cause detrimental effects^22^. Reference to the negative association of fasting insulin levels and insulin resistance with pancreatic β-cell function found by Zhang *et al.*^23^. We observed that, compared with Silky chickens, broiler chickens showed insulin resistance with higher fasting serum insulin concentrations, and impaired homeostasis of both blood glucose and the circulatory insulin in response to exogenous insulin. In contrast, Silky chickens showed stronger glucose homeostasis regulation than AA broilers, which was consistent with their higher IR levels in the basal state in the pancreas and the stronger serum insulin homeostasis regulation in response to exogenous insulin. It showed that the difference in the regulation of blood glucose homeostasis between two breeds may be related to their pancreatic IR levels and fasting insulin levels at the basal state and their different dynamic responses to exogenous insulin. Similarly, hyperphagic low weight chickens successfully return to initial blood glucose levels at 180 min after insulin injection (80 μg/kg weight), whereas hypophagic obese high weight chickens failed to do this^24^. Results suggest that low body weight chickens should have better glucose regulation ability in response to exogenous insulin perturbation.

In mammals, URIC may affect blood glucose levels, disturbing glucose metabolism and insulin sensitivity^25^. Insulin secretory defects have been found to be associated with human chronic kidney disease arising from elevated circulating levels of urea that may increase islet protein O-GlcNAcylation and impair glycolysis^26^. Here, we also observed that blood glucose was positively correlated with circulating urea and URIC. Birds had relatively high plasma glucose levels, as well as high metabolic rates and body temperatures, but they are much longer-lived than mammals with equivalent body mass^27^. This phenomenon was thought to be related to a process likely to occur through endogenous antioxidant mechanisms, such as those involving URIC, a potent antioxidant that ameliorates oxidative stress^28^. In this study, we found that at 120 min of insulin treatment, the CHOL concentration in the serum of Silky fowls increased significantly, and the LDLD responsible for transporting CHOL also increased to a certain extent, but this phenomenon was not observed in broilers. We suspect that Silky fowls may use more fat breakdown to provide energy to maintain the body needs, which may also be part of the reason for the rapid blood glucose recovery.

In mammals, liver and skeletal muscle are the main insulin sensitive tissues. Skeletal muscle is responsible for a significant fraction of glucose uptake from the blood stream in the insulin-stimulated state^29^. More than 60% of blood glucose is absorbed by skeletal muscle^30,31^. And in growing broiler chickens, with the rapid increase of skeletal muscle mass, it is likely that glucose uptake by the skeletal muscles increases in importance^32^. The insulin cascade appears to respond normally in chicken liver but to be refractory in chicken muscle^11,33^, lack of GLUT4 might be one reason for the unorthodox glucose homeostasis in chickens. IR is in an early stage in the insulin signaling cascade. In accordance with findings from Dupont *et al*.^33^, in the present study, insulin stimulation did not result in a significant change in IR mRNA levels in the pectoralis of both broiler and Silky chickens.

Recent findings in mammals suggest that, in addition to GLUT4, GLUT12 may be a second insulin-sensitive glucose-transporter^10^. In this research, GLUT12 was predominantly expressed in birds’ skeletal muscle, as previously reported^11^. Moreover, IR mRNA level showed a strong correlation with GLUT12 level in the PM tissue (r = 0.5116). These suggested that GLUT12 maybe an important insulin-sensitive transporter and function as the major glucose transporter in compensation for the lack of GLUT4 in chicken skeletal muscle tissue, and that chicken GLUT12 may regulate blood glucose in birds through the IR signaling cascade pathway, as occurs in mammals.

In chickens, similarly to mammalian species, there is abundant GLUT2 expression in the liver and pancreas^34,35^. GLUT2 gene expression has been shown to be concerned in the regulation of glucose metabolism in the liver^36,37^. In this study, GLUT2 mRNA level was down-regulated at 120 min and 240 min in the liver post insulin injection both in broilers and Silky fowls. Zhang *et al*. have also found that exogenous insulin decreases the mRNA abundance of IR and GLUT2 (60 min after intraperitoneal injection) in the liver in broilers^14^. Chicken liver GLUT2 mRNA was also down-regulated in day 16 broiler embryos and newly hatched chicks after insulin injection^38^. The down regulation of GLUT2 in the liver in fowls may be a compensatory mechanism to prevent further utilization of glucose in the liver during insulin-induced hypoglycemia that sustains hyperglycemia even in a fasting state. In addition, our research showed that GLUT2 was more insulin sensitive in broilers than in Silky chickens, in multiple tissues including the liver, pancreas and OB.

As a metabolic sensor of brain insulin and glucose concentrations^17^, the OB tissue of broilers was more sensitive to exogenous insulin, where GLUT2, NPY and IR mRNA levels were significantly changed. NPY is the most potent orexigenic peptide found in the brain; it decreases latency to eating, increases motivation to eat and delays satiety by augmenting meal size^39^. Sindelar *et al*. found after induction of a moderate hypoglycaemia by insulin, the feeding response is much larger in normal mice than in mice lacking NPY^40^. In our research, we found that NPY was highly expressed in the OB tissue of broilers in the basal state, whereas insulin stimulation resulted in aggressive NPY up-regulation in the OB tissue of Silky fowls but not broilers at 240 min. The level change of NPY in the OB tissue was in accordance with the appetite action in two breeds, we observed that Silky chickens had better appetite recovery after 120 min of insulin injection, and more Silky chickens began to forage at 120 min after refeeding.

## Conclusions

Silky fowls presented greater blood glucose recovery than AA broilers in the insulin resistance tests. The strong ability to regulate glucose homeostasis is in agreement with their higher IR levels in the basal state and stronger serum insulin homeostasis regulation response to exogenous insulin. In contrast, broilers showed resistance with higher fasting serum insulin concentrations, and impaired regulation of blood glucose homeostasis and the serum insulin homeostasis response to exogenous insulin. The difference between the two breeds in the regulation of blood glucose homeostasis may be related to their IR levels in the pancreas in the basal state and after exogenous insulin stimulation. GLUT2, GLUT12, NPY and IR are predominantly expressed in the liver, PM, OB and pancreas, respectively, and the genes had relatively stronger insulin sensitivity in the tissue where they were predominantly expressed. We observed a positive correlation between IR and GLUT12 mRNA expression not only in the PM tissue but also in all detected samples, which supports that chicken GLUT12 in skeletal muscle acts as an insulin sensitive transporter similar to GLUT4 in mammals, and suggests that chicken GLUT12 may regulate blood glucose in birds through the IR signaling cascade pathway.

## Materials and methods

### Animals

Female AA broilers and Silky fowls were reared in with free access to water and a conventional balanced diet, which was formulated according to the nutritional standards for broilers recommended by the NRC (Nutrient Requirements for Poultry, 1994) in mash form until 47 days of age. The research protocol was approved by the Animal Care and Use Committee of Henan Agricultural University (Zhengzhou, China). The room temperature was maintained at 33–35 °C in the first week, and decreased by 2–3 °C per week there after until 24 °C and keep this temperature; humidity and ventilation were controlled. Chickens were exposed to a 24-h light regimen in the first week and then maintained for 23 h.

### Insulin tolerance tests and sample collection

At 47 days of age, after 12 h fasting (free access to water) chickens with similar body weight (close to the average weight) of AA broilers (average weight = 1.86 kg) and Silky fowls (average weight = 0.75 kg) were randomly divided into the insulin group (n = 12/breed) and phosphate buffered saline (PBS) group (n = 12/breed), respectively. The insulin groups received an injection of insulin (80 μg/kg BW), and the PBS control groups were injected with an equal volume of PBS. Blood glucose was measured at 0, 15, 30, 60 and 120 min (after insulin/PBS injection) via the wing vein with a handheld glucometer (ACCU-CHEK Performa, Roche, Germany). Fowls were given feed at 120 min after insulin injection for 2 h, and blood glucose concentrations were measured again at 240 min post injection. Before PBS/insulin injection, six chickens from the Silky and broiler groups were sacrificed after 12 h fasting (denoted 0 min). In addition, the insulin group chickens were killed at 120 min and 240 min after insulin injection (n = 6 for each time point/breed), and their blood and tissues including the PM, OB, pancreas and liver were collected. Tissues were snap-frozen in liquid nitrogen and stored at −80 °C until use. The collected blood samples were allowed to stand at room temperature (20–25 °C) for 2–3 h after collection. After naturally precipitated, the serum was separated under 1500–2000 × g/min for 10 min.

The collected serum was divided into two 1.5 ml centrifuge tubes for the determination of serum insulin levels and other serum biochemical indexes, and stored at −20 °C until use. The content of serum insulin was determined through an insulin enzyme linked immunosorbent assay according to the manufacturer’s instructions (Nanjing Jiancheng Bioengineering Institute, Nanjing, China). An automatic biochemistry analyzer (CX4/Pro, Beckman, Brea, CA, USA) was used to determine other serum metabolites including urea, uric acid (URIC), cholesterol (CHOL), triglyceride, high density lipoprotein (HDLD) and low density lipoprotein (LDLD).

### Total RNA isolation and Real-time PCR

Total RNA was extracted from liver, PM, OB and pancreas using Trizol reagent (Mei5 Biotechnology Co., Ltd, Beijing, China). The concentration and quality of the isolated RNA were determined by a spectrophotometer (Thermo NanoDrop One, America) and agarose-gel electrophoresis, respectively. The gel electrophoresis diagram is shown in supplementary file. The cDNA was synthesized using a PrimeScrip RT reagent Kit with gDNA Eraser (Perfect Real Time, TaKaRa Biotechnology, Co., Ltd., Dalian, P. R. China) in a 20 µl reaction containing 1000 ng of total RNA with random primers and oligo dT primer according to the manufacturer’s instruction. The real-time PCR primers for amplifying chicken GLUT2, GLUT12, NPY, IR and β-actin (Table 1) were designed with NCBI Primer-Blast (https://www.ncbi.nlm.nih.gov) and synthesized by Sangon (Sangon Biological Engineering Technology & Service Co., Ltd, Shanghai, China). The β-actin gene was used as an internal control to normalize the individual RNA input. Real-time quantitative PCR was performed in 10 µl reactions contained SYBR Green Master Mix (Mei5 Biotechnology Co., Ltd, Beijing, China), forward and reverse primers (0.1 μM each) and 1 µl cDNA in a BioRad CFX96 (BioRad, America). Real-time PCR reactions were performed in triplicate for each sample under the following conditions: initial denaturation at 95 °C for 5 min, followed by 38 cycles of 95 °C for 30 s, 60 °C for 30 s, and 72 °C for 30 s; and 72 °C for 1 min. Melting curve analyses were performed after all PCR reactions to ensure amplicon Specificity. Real-time PCR data were analyzed with the 2^−∆∆CT^ method, where ∆C_T_ = C_T target gene_ − C_T β-actin_, and ∆∆C_T_ = ∆C_T target sample_ − ∆C_T calibrator_ (maximum average C_T_ value for multiple breeds and multiple tissues and multiple times)^41^.

### Statistical analysis

Data were analyzed in SPSS 18.0 and expressed as the means ± standard error. One-way ANOVA was used to analyze changes in blood glucose and gene mRNA abundance difference in a certain tissue in the basal state between broilers and Silky fowls. In addition to one-way ANOVA, two-way ANOVA was used to analyze the association between breed and time effect on serum metabolites and insulin level and genes mRNA abundance. If the main or interaction effect was significant, the data were analyzed again by one-way ANOVA. The correlations of serum glucose with serum biochemical indexes, and expression of some two genes were analyzed with Pearson correlation with two-tailed tests. *P* < 0.05 was considered significant.

## Supplementary information

Supplementary information.

## References

[1] Henquin, J. C. J. Regulation of insulin secretion: a matter of phase control and amplitude modulation. *Diabetologia*. **52**, 739–751. 10.1007/s00125-009-1314-y 19288076

[2] Zhao, N. *et al*. Apolipoprotein E4 Impairs Neuronal Insulin Signaling by Trapping Insulin Receptor in the Endosomes. *Neuron*. **96**, 115–129.e115. 10.1016/j.neuron.2017.09.003 PMC5621659 28957663

[3] Yusaku, Nakabeppu (2019). Origins of Brain Insulin and Its Function. Diabetes Mellitus..

[4] Akiba Y (1999). Persistent hypoglycemia induced by continuous insulin infusion in broiler chickens. British Poultry Science..

[5] Uldry M, Thorens B (2004). The SLC2 family of facilitated hexose and polyol transporters. Pflügers Archiv..

[6] Tokushima Y, Takahashi K, Sato K, Akiba Y (2005). Glucose uptake *in vivo* in skeletal muscles of insulin-injected chicks. Comparative Biochemistry and Physiology Part B: Biochemistry and Molecular Biology..

[7] Zhao JP (2012). Altered gene and protein expression of glucose underlies dexamethasone inhibition of insulin-stimulated glucose uptake in chicken muscles. J. Anim. Sci..

[8] Watson RT, Pessin JEJ (2001). Intracellular Organization of Insulin Signaling and GLUT4 Translocation. Recent Progress in Hormone Research..

[9] Carver FM, Shibley IA, Pennington JS, Pennington SN (2001). Differential expression of glucose transporters during chick embryogenesis. Cellular and Molecular Life Sciences Cmls..

[10] Coudert E (2015). Phylogenesis and Biological Characterization of a New Glucose Transporter in the Chicken (Gallus gallus), GLUT12. PLoS One..

[11] Dupont J, Tesseraud S, Simon J (2009). Insulin signaling in chicken liver and muscle. General Comparative Endocrinology..

[12] Franssens L (2016). The effect of insulin on plasma glucose concentrations, expression of hepatic glucose transporters and key gluconeogenic enzymes during the perinatal period in broiler chickens. Gen. Comp. Endocrinol..

[13] Leturque A, Brotlaroche E, Le GM, Stolarczyk E, Tobin V (2005). The role of GLUT2 in dietary sugar handling. J. Physiol. Biochem..

[14] Zhang W, Sumners LH, Siegel PB, Cline MA, Gilbert ER (2013). Quantity of glucose transporter and appetite-associated factor mRNA in various tissues after insulin injection in chickens selected for low or high body weight. Physiol. Genomics..

[15] Coudert E (2018). Expression of glucose transporters SLC2A1, SLC2A8 and SLC2A12 in different chicken muscles during ontogenesis. J. Anim. Sci..

[16] Proszkowiec-Weglarz M (2017). Insulin immuno-neutralization decreases food intake in chickens without altering hypothalamic transcripts involved in food intake and metabolism. Poult. Sci..

[17] Tucker K (2010). The Olfactory Bulb: A Metabolic Sensor of Brain Insulin and Glucose Concentrations via a Voltage-Gated Potassium Channel. Results Probl. Cell Differ..

[18] Campbell PJ, Carlson MG, Hill JO, Nurjhan NJ (1992). Regulation of free fatty acid metabolism by insulin in humans: Role of lipolysis and reesterification. American Journal of Physiology..

[19] Berliner JA, Frank HJL, Karasic D, Capdeville MJ (1984). Lipoprotein-induced Insulin Resistance in Aortic Endothelium. Diabetes..

[20] Sosenko JM, Breslow JL, Miettinen OS, Gabbay KHJ (1980). Hyperglycemia and plasma lipid levels. A prospective study of young insulin-dependent diabetic patients. New England Journal of Medicine..

[21] Adeli K, Taghibiglou C, Iderstine SCV, Lewis GFJ (2001). Mechanisms of Hepatic Very Low-Density Lipoprotein Overproduction in Insulin Resistance. Trends Cardiovasc. Med..

[22] Vargas, E. & Carrillo Sepulveda, M. A. In *StatPearls* (StatPearls Publishing, 2019). 30726007

[23] Zhang Z, Wang J, Wang HJ (2018). Correlation of blood glucose, serum chemerin and insulin resistance with NAFLD in patients with type 2 diabetes mellitus. *Experimental and Therapeutic*. Medicine..

[24] Sumners LH (2014). Chickens from lines artificially selected for juvenile low and high body weight differ in glucose homeostasis and pancreas physiology. Comp. Biochem. Physiol. A Mol. Integr. Physiol..

[25] Wardhana W, Rudijanto A (2018). Effect of Uric Acid on Blood Glucose Levels. The Biochemical journal..

[26] Franssens L (2016). The effect of insulin on plasma glucose concentrations, expression of hepatic glucose transporters and key gluconeogenic enzymes during the perinatal period in broiler chickens. General & Comparative Endocrinology..

[27] Holmes DJ, Flückiger R, Austad SN (2001). Comparative biology of aging in birds: an update. Exp. Gerontol..

[28] Braun EJ, Sweazea KL (2008). Glucose regulation in birds. Comparative Biochemistry and Physiology Part B..

[29] Efendić, S. Pathogenesis of NIDDM. **4**, 8–10, (1988). 10.1016/0168-8227(88)90005-8 3042340

[30] Geng T (2018). H19 lncRNA Promotes Skeletal Muscle Insulin Sensitivity in Part by Targeting AMPK. Diabetes..

[31] Guo X (2019). Panax notoginseng saponins alleviate skeletal muscle insulin resistance by regulating the IRS1–PI3K–AKT signaling pathway and GLUT4 expression. FEBS Press..

[32] Kono T (2005). Characterisation of glucose transporter (GLUT) gene expression in broiler chickens. Br. Poult. Sci..

[33] Dupont JL, Dagou C, Derouet M, Simon J, Taouis M (2004). Early steps of insulin receptor signaling in chicken and rat: apparent refractoriness in chicken muscle. Domest. Anim. Endocrinol..

[34] Wright E, Turk E (2004). The sodium/glucose cotransport family SLC5. Pflugers Arch..

[35] Shannon BM, Christianna H, Xiaofei W (2017). Avian and Mammalian Facilitative Glucose Transporters. Microarrays..

[36] Hall JR, Short CE, Driedzic WR (2006). Sequence of Atlantic cod (Gadus morhua) GLUT4, GLUT2 and GPDH: developmental stage expression, tissue expression and relationship to starvation-induced changes in blood glucose. J. Exp. Biol..

[37] Weinstein, S. P., O’Boyle, E., Fisher, M. & Haber, R. S. J. Regulation of GLUT2 glucose transporter expression in liver by thyroid hormone: evidence for hormonal regulation of the hepatic glucose transport system. *Endocrinology*. **2** (1994). 10.1210/endo.135.2.8033812 8033812

[38] Franssens L, Buyse J, Decuypere E, Everaert N (2014). Relationship between glucose and pancreatic hormones during the embryonic and postnatal phase in chickens. Avian Biology Research..

[39] Beck B (2006). Neuropeptide Y in normal eating and in genetic and dietary-induced obesity. Philosophical Transactions Biological Sciences..

[40] Sindelar DK (2004). Neuropeptide Y Is Required for Hyperphagic Feeding in Response to Neuroglucopenia. Endocrinology..

[41] Rao X, Huang X, Zhou Z, Lin X (2013). An improvement of the 2ˆ(–delta delta CT) method for quantitative real-time polymerase chain reaction data analysis. Biostat Bioinforma Biomath..

