# Supplementary material for: Dynamic changes of blood glucose, serum biochemical parameters and gene expression in response to exogenous insulin in Arbor Acres broilers and Silky fowls
Source: Sci Rep. 2020 Apr 21;10:6697. doi: 10.1038/s41598-020-63549-9 (PMC7174311; doi:10.1038/s41598-020-63549-9)
Supplement: Supplementary file 1 — Supplementary information. [file 41598_2020_63549_MOESM1_ESM.pdf]

Dynamic changes of blood glucose, serum biochemical parameters and gene expression in response to exogenous insulin in Arbor Acres broilers and Silky fowls

Jiefei Ji, Yafei Tao, Xiangli Zhang, Jiajia Pan, Xinghao Zhu, Huanjie Wang, Pengfei Du, Yao Zhu, YanQun Huang*,Wen Chen

College of Animal Science, Henan Agricultural University, Zhengzhou, Henan, P. R. China

*Correspondence: hyanqun@aliyun.com

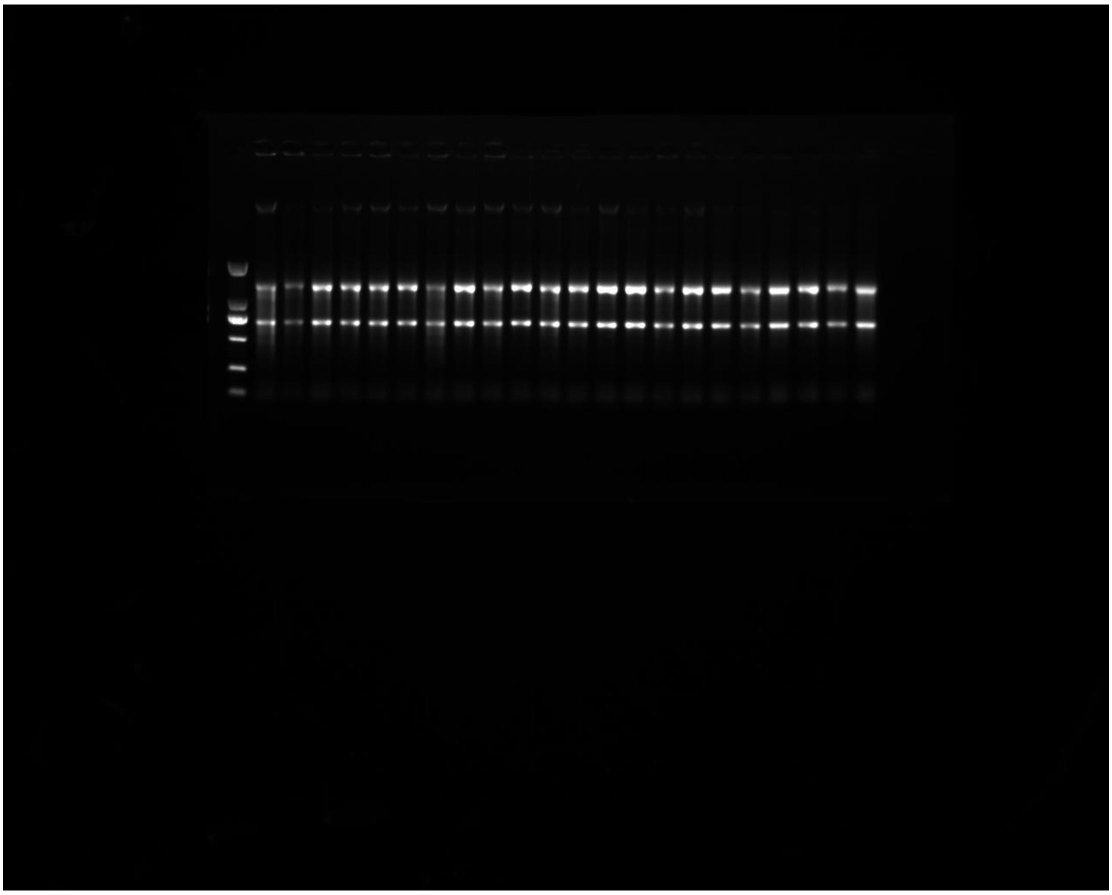

Figure S1 RNA gel electrophoresis.
